# Exercise, addiction and motivation: the development of a motivation scale in extreme sports as a sport of challenge with nature

**DOI:** 10.3389/fpsyg.2025.1651139

**Published:** 2025-12-31

**Authors:** İzzet Karakulak, Özge Yavaş, Mehmet Veysi Bora, Ender Eyuboğlu, Cem Sinan Aslan, Ahmet Aydemir, Sırrı Cem Dinç, Ömür Fatih Karakullukçu, Faik Öz, Fatma Neşe Şahin

**Affiliations:** 1 Faculty of Sport Sciences, Mardin Artuklu University, Mardin, Türkiye; 2 Faculty of Sport Sciences, Bartın University, Bartın, Türkiye; 3 Faculty of Sport Sciences, Burdur Mehmet Akif Ersoy University, Burdur, Türkiye; 4 Faculty of Sport Sciences, Trabzon University, Trabzon, Türkiye; 5 Ministry of National Education, Ankara, Türkiye; 6 Faculty of Sport Sciences, Avrasya University, Trabzon, Türkiye; 7 Faculty of Sport Sciences, Ankara University, Ankara, Türkiye

**Keywords:** extreme sports, adaptive sports, addiction, motivation, sports psychology

## Abstract

In the extant literature, the concepts of motivation and addiction have been employed to elucidate how a considerable number of unpleasant experiences for extreme sports participants can eventually become rewarding. The objective of this study was to develop a scale to measure the behavioral addiction levels of extreme sports athletes. A plethora of addiction and motivation scales (MS) exists; however, none of them are measurement tools that can assess emotional responses and simultaneously address motivation and addiction. Moreover, they are not specific to extreme sports participants. The present study comprised 1,073 participants engaged in extreme sports and incorporated psychometric analyses encompassing a literature review, expert review, exploratory and confirmatory factor analysis. The MS factor structure was determined using the Principal Axis Factoring (PAF) method. The analysis yielded a three-factor structure comprising 16 items, accounting for 63.00% of the total variance. In order to validate the structure, first- and second-order factor analysis was performed. The fit indices obtained at both levels were consistent with the acceptable limits specified in the literature. In the final results, 16 items related to three factors, namely “hedonic deprivation,” “tolerance,” and “hedonic opposition,” were validated. Furthermore, the AVE, CR, and √AVE values were calculated. The analysis results indicated that each sub-dimension in the measurement model exhibited a distinct structure and fulfilled the established validity criteria. Cronbach’s Alpha and Split-Half coefficients were calculated to ascertain the reliability of the measurement. The floor and ceiling effects of the MS were also examined. Finally, ROC analysis was employed to evaluate the measurement tool. The findings of this research indicate that the MS, which has been developed and comprises 16 items and a three-factor structure, is a valid and reliable instrument capable of simultaneously addressing motivation and addiction in the context of extreme sports. The scale is of significance as it is the inaugural quantitative measurement tool with the capacity to assess addiction. In conclusion, the MS provides a novel, theory-based contribution to the literature by integrating motivation and behavioral addiction within a single, psychometrically validated framework specific to extreme sports.

## 1 Introduction

It is evident that human beings are capable of engaging in warfare without any apparent benefit, driven solely by instinctual impulses. In a similar vein, although games and sports may be regarded as energy-consuming, “aimless” activities, their fundamental purpose is to facilitate the development and mastery of behaviors essential for survival in a risk-free environment. Consequently, games and sports function as a form of “practice battle” or challenge, preparing individuals for real-life threats (Gat, 2010). Early humans discovered methods of artificially inducing feelings of excitement and happiness, eschewing activities in favors of the use of narcotic substances. The effects of narcotics such as ecstasy are characterized by an elevation in hormonal levels, including adrenaline, serotonin and dopamine. This hormonal surge is often accompanied by a sense of euphoria, a reduction in sensitivity to pain and fatigue, and an overall sense of well-being. This provides a rationale for the utilization of such narcotics in preparation for war by the Hashashins, the feared assassins of their era (Gat, 2010).

In the context of nature-based activities, euphoric behaviors typically manifest during periods of excessive physical exertion and are frequently associated with a sense of struggle (Derelioğlu et al., 2025). It is evident that forms of “adventure recreation” which motivate individuals to participate in activities in natural settings have now become recognized as “extreme” sports (Burgin and Hardiman, 2012). Carnicelli-Filho et al. (2010) describe these sports as pushing the existing limits of risk for the sake of excitement, overcoming fear caused by speed, gravity, or height to stimulate an adrenaline “rush.” In recent decades, a plethora of sporting activities have emerged which can be categorized under the umbrella of ‘extreme sports’. These include extreme sports, nature sports, adventure sports, risk sports, gravity sports, adventure recreation, outdoor education, outdoor recreation, nature tourism, ecotourism, adventure tourism, ‘whiz sports’, ‘free sports’, ‘adventure sports’, ‘lifestyle sports’, ‘action sports’, ‘alternative sports’, and ‘extreme sports’. Despite minor differences in meaning, these terms have many similarities (Buckley, 2012 Melo and Gomes, 2017; Brymer and Houge-Mackenzie (2017); Rinehart, 2016; Amador Durán-Sánchez et al., 2020).

In the vast majority of activities undertaken in a natural environment, the inherent challenges posed by nature to humans are examined (Eisen et al., 2024). In contrast to other combat sports, such as war or competition, this struggle is not a direct confrontation between two parties, but rather a challenge to the natural order of things (Brymer and Gray, 2010). From this standpoint, engaging in extreme sports that require a high degree of physical exertion and risk, such as boxing or wrestling, can be seen as a form of internal conflict, where the individual is pitted against their own inner struggles and limitations (Oztas and Vural, 2023). The concept of complete union with nature, that is, existing in nature, is, in fact, a state of being that is already present within oneself (Brymer and Gray, 2010). Furthermore, the pursuit of novel experiences, the exhilaration derived from adventure, and the challenge of both perceived and actual risks are cited as motivating factors (Melo et al., 2020; Melo and Gomes, 2017). Theories have been advanced to explain participation motivation, in addition to the search for risk or adrenaline. These theories have been supported by a series of analytical frameworks, including working at the edge, thrill seeking, psychoanalysis, neotribalism or subculture formation, and masculinity theory (Brymer et al., 2017).

Participants are described as thrill seekers who are risk-taking and adrenaline-dependent (Lambton, 2000; Baker and Simon, 2010; Palmer, 2004; Robinson, 2004) and death-seeking (Slanger and Rudestam, 1997). Furthermore, such behaviors are described as pathological, unhealthy, and socially unacceptable, deviant behaviors (Elmes and Barry, 1999; Monasterio, 2007; Pain and Pain, 2005; Self et al., 2007). However, it has been asserted that traditional theory-focused perspectives should be critiqued, as they disregard interaction with nature and the positive outcomes obtained (Brymer, 2010; Brymer and Schweitzer, 2013), do not reflect the experiences of participants, and are formulated by non-participants (Brymer and Houge Mackenzie, 2017; Mackenzie and Brymer, 2020). Participation in extreme sports is increasingly understood through psychological frameworks that emphasize internal motivation, emotional regulation, self-fulfillment and identity development rather than simple risk-seeking or adrenaline-based explanations. The multidimensional nature of these experiences reflects complex motivational dynamics driven by personal challenge, flow experience, autonomy and a search for meaning (Hornby et al., 2024; Martinho et al., 2024). This perspective highlights that extreme sports represent a context in which individuals test their psychological limits, regulate emotional states and experience profound well-being, suggesting that the motivation to participate goes beyond physical performance and risk-based narratives (Terzioğlu and Çakır Çelebi, 2024).

Contrary to popular belief, recent studies have shown that participation in extreme sports is not related to risk-taking, excitement, or the need for adrenaline (Brymer, 2010). The predominant rationale for the reinforcement of this perception within society is the frequent labeling of participants as adrenaline junkies by popular media (Storry, 2003; Pain and Pain, 2005; Salassa and Zapala, 2009; Brymer and Schweitzer, 2013). Participants characterize these sporting activities as both exhilarating and transformative experiences. These experiences are often compared to those of individuals who use drugs. Participants in this study explain their experiences in terms of addiction, stating that they suffer when they do not engage in the activity to a sufficient extent (Celsi et al., 1993; Price and Bundesen, 2005; Willig, 2008; Buckley, 2012). As with drug and non-drug addictions, the emergence of strong urges or cravings for extreme sports is defined in the psychopharmacology literature as a subjective motivational state characterized by an intense desire for the effects of a drug (Grant et al., 2010). However, the situation is not only related to pharmacological or narcotic substances, as is the case with adrenaline, but is a direct addiction to the behavior exhibited to experience that environment. Indeed, as Buckley (2012) reported, more comprehensive research could be conducted by conceptualizing extreme sports as behavioral addictions.

It is important to note that behavioral addictions are not dependent on a physical substance. They are difficult to control and are characterized by continued actions despite negative consequences (Griffiths, 2005; American Psychiatric Association, 2013). Internet usage (Griffiths, 2005), social media (Griffiths, 2013), smartphones (Lin et al., 2014), gambling, shopping, exercise, gaming, sexuality, and even participation in extreme sports are all examples of behavioral addictions. As demonstrated in the works of Grant et al. (2010), Potenza (2006), and Weinstein et al. (2014), as well as Olsen (2001) and Leeman and Potenza (2013),

Withdrawal syndrome has been identified as an indicator of addiction, and it is defined as the set of feelings experienced when a substance or behavior that causes addiction is discontinued. This phenomenon may manifest as physiological and/or psychological distress (Farrell, 1994; West and Gossop, 1994). Withdrawal syndrome is characterized by a decrease in interest or pleasure in previously rewarding stimuli and is associated with anhedonia, or a negative mood (Sekine et al., 2003; Zijlstra et al., 2008). Anhedonia, movement, reward, and addictions have been primarily associated with dopamine. Decreased dopamine levels have been demonstrated to be associated with increased anhedonia or emotional indifference (De Vries et al., 2022). Franken et al. (2006) conducted a study among skydivers and posited the hypothesis that individuals engaging in high-risk extreme sports experience elevated levels of anhedonia. The researchers further suggested that, akin to substance addicts, extreme athletes may encounter anhedonia during periods of abstinence from their activities. Should the withdrawal symptoms exhibited by extreme athletes be likened to those experienced by drug users, then participation in such activities could serve as a conduit for drug users to divert their attention from drug use and alleviate withdrawal symptoms. In the extant literature, researchers posit that the risk and physiological arousal inherent in participating in such sports may satisfy the cravings of drug users, and therefore such risk-taking activities should be included in addiction treatment programs (Franques et al., 2003; Slaght et al., 2004; Ersche et al., 2010). It has been hypothesized that these activities may function as a mood stabilizer, thereby facilitating the recovery process for individuals who use drugs. In this regard, it has been demonstrated that these activities may mitigate negative effects (Woodman et al., 2008; Woodman et al., 2010). It is hypothesized that such measures will facilitate the comparison of behavioral addiction levels among extreme sports participants and a more profound understanding of the similarities between drug and non-drug addictions. In this regard, there is a need to develop a reliable and valid “motivation” scale based on the Opposite Process Theory in order to investigate whether the motivation to participate in extreme sports is the result of behavioral addiction.

### 1.1 Opponent process theory

Its first foundations were laid under the name The Theory of Opponent Colors, and the model was studied by Johann Wolfgang von Goethe in his 1810 book The Theory of Colors. It is about the physiological effect of contrasting colors. Refined by the German physiologist Ewald Hering, he suggested that the way we see colors is based on a system of contrasting colors (Comer, 2015). He emphasizes that red, yellow, green and blue colors cannot be perceived as a mixture of a color, that is, they can be perceived as either red or green, and never greenish red. In 1957, Leo Hurvich and Dorothea Jameson provided quantitative data for Hering’s contrasting-color theory. In 1967 Rod Grigg extended the model to reflect a wide variety of opponent processes in biological systems. The Opponent Color Theory, which can be adapted to a wide variety of situations, was first used by Luria (1932) in the study of an acute real-life event to measure the response to conflict and stress, under the name “Rival Process Theory.” Subsequently, following the tradition of Luria’s work by Epstein and Fenz (1962, 1965) and Fenz and Epstein (1967) researchers examined the skydiving experiences of inexperienced ones (Comer, 2015).

The opponent-process theory, introduced to explain motivational and emotional dynamics, is actually a psychological and neurological model that explains a wide variety of behaviors, including color vision. The model extended by Solomon and Corbit (1973, 1974) and Solomon (1977, 1980a, b) of the “emotional or hedonic opposition that occurs when a negative reinforcer is presented. Then removed,” discussed in the opponent-process theory, comes from the research report of Epstein and Fenz (1962, 1965) on the emotional responses of military paratroopers. Solomon and Corbit (1973, 1974) and Solomon (1977, 1980a, b) handled the theory to explain the concepts of emotion, addiction, and motivation. The theory basically states that the process with a pleasant or unpleasant emotional balance is followed by a secondary “opponent process.” This opponent process kicks in after the primary process is stopped. With repeated exposure, the primary process is weakened while the opponent process is strengthened. Solomon’s most important contribution to the theory has been his research findings on motivation and addictive behavior with paratroopers. In the study of Solomon and Corbit (1973, 1974) and Solomon (1977, 1980a, b) in which they observed the emotional changes of free skydivers with this theory, the reason for researchers to choose free skydivers is that they experience extreme hedonic experiences, and these experiences cause emotional changes in them. According to the opponent process theory performed on skydivers, while skydivers experience their first freefall, they may show different reactions during the fall (before the parachute opens) (their eyes may swell, their lips may retract, they may yell, their body will bend forward or even involuntarily urinate). Because autonomic nervous systems are faced with a high stimulus at that moment. After the parachute is opened, there is fear until it lands, and it has been observed that in the first minutes after landing in the safe area, the skydivers were confused, dull-eyed, and did not talk much, but after a few minutes, they started social interaction and chatting. The reaction takes about 10 min. After many parachute jumps, responses such as fear are often undetectable. Instead, skydivers appear nervous, eager, or excited and they are reported to experience “excitement” during freefall. After a safe landing, withdrawal syndrome occurs and the person experiences jumping, shouting, rapidly changing social interactions, loud speech, and general enthusiasm. This period often described as cheering, gradually decreases over time, but usually lasts for 2–3 h. The activity level rises with the desire to repeat a situation that the parachutist is used to after the first jump, but initially frightening. If we summarize the model revealed by Solomon and Corbit (1973, 1974), Solomon (1977, 1980a, b); those who experienced the first jump experienced a great fear in their first jump and after a while, this fear turned into great relief. With repeated jumps, skydivers’ fear of jumping decreased, and their pleasure after jumping increased (Solomon and Corbit, 1973, 1974; Solomon, 1977, 1980a, b).

### 1.2 Opponent process theory and addiction in extreme sports

A theoretical framework for understanding the relationship between opponent process theory and addiction in extreme sports.

As postulated by opponent process theory, the human psyche is capable of experiencing a duality of emotions, including pairs such as happiness and sadness, fear and relief, and pleasure and pain. In the event of the emergence of one of these, the other is temporarily suppressed. Nevertheless, these opposing emotions can also resurface with great intensity, thereby impeding or preventing the initial emotion from manifesting. Therefore, the activation of one emotion invariably precipitates its antithesis, resulting in a dynamic interplay between these two discrete entities. While this can be used to explain motivational and addictive behaviors to a certain extent, it can also explain why people find enjoyment in horror movies or other exciting activities, such as skydiving. In other words, it can explain why situations that are unpleasant for many people can be rewarding for some (Solomon and Corbit, 1973).

The extant literature on the subject of motivation and addiction in extreme sports is scant. One such piece of evidence is the Opponent Process Theory proposed by Solomon and Corbit (1973), which forms the theoretical basis of the research. Utilizing this theoretical framework, Solomon and Corbit (1973) conducted a study that observed the emotional changes experienced by skydivers. According to Solomon and Corbit (1973, 1974) and Solomon (1977, 1980a, b), emotional or hedonic processes are involved in every case of acquired motivation. When acquiring motivation is defined, three emotional or hedonic phenomena are present in every case. These three emotional or hedonic phenomena can be defined as emotional or hedonic opposition, emotional or hedonic tolerance (emotional and hedonic habituation/tolerance), and emotional or hedonic deprivation (emotional and hedonic deprivation/abstinence). These three emotional or hedonic phenomena are characteristic of many acquired drives and have been likened to the phenomenon of opium addiction. In the context of an examination of the emotional process associated with opium use, the initial administration of opium results in the experience of a pronounced surge of pleasure, which is commonly referred to as a “rush” in state A. This is subsequently followed by a less pronounced decline in intensity, often characterized by the sensation of euphoria, which serves as the initial indication of habituation. In this paradigm, the initiation of substance intake functions as a “positive reinforce.” Subsequently, after the substance’s effects wear off, state B emerges, a disincentive desire state known as withdrawal syndrome. In the final stage, the craving gradually disappears. In such circumstances, there are two motivational events capable of reinforcing the behavior: the onset of state A and the disappearance of state B. However, if the individual repeatedly administers the drug doses, two related changes in sensation occur:

- The rush is no longer experienced, and euphoria is generally absent (loss of euphoria).
- The withdrawal syndrome is characterized by a marked intensification of symptoms, manifesting in both physiological and psychological domains. Notably, the duration of the syndrome is significantly prolonged. Thus, the positive reinforcing power is diminished, while the negative reinforcing power is augmented and persists for a more protracted duration. The motivation for drug use undergoes a transition from positive to negative control as the doses escalate. It has been demonstrated that, in response to repeated administration of the drug, an individual may develop a tolerance to its effects, and, concomitantly, a heightened intolerance to its withdrawal or absence (Solomon and Corbit, 1973, 1974; Solomon, 1977, 1980a, b). The theory must be qualified with the specificity assumption at the point at which state B is the rival of state A, and the fastest and most effective way out of state B is to use the substance that directly produces state A. This is the case with addictive drugs. It can thus be posited that the behavior of obtaining and using the substance that triggers state A will be strongly reinforced, given that it produces state A while simultaneously ending state B. This will result in state B becoming even stronger. Consequently, it is imperative to augment the quantity of the substance in order to ensure that the sum of (a-b) exceeds zero or surpasses the initial level. This phenomenon is known as drug tolerance. Increasing the dosage has been shown to strengthen all associated behaviors (Jones et al., 2020). Furthermore, with more frequent use, state B is known to become even stronger (Smith, 2021). This dynamic is conceptualized as the cycle of addiction (Solomon and Corbit, 1974).

**Figure 1:**
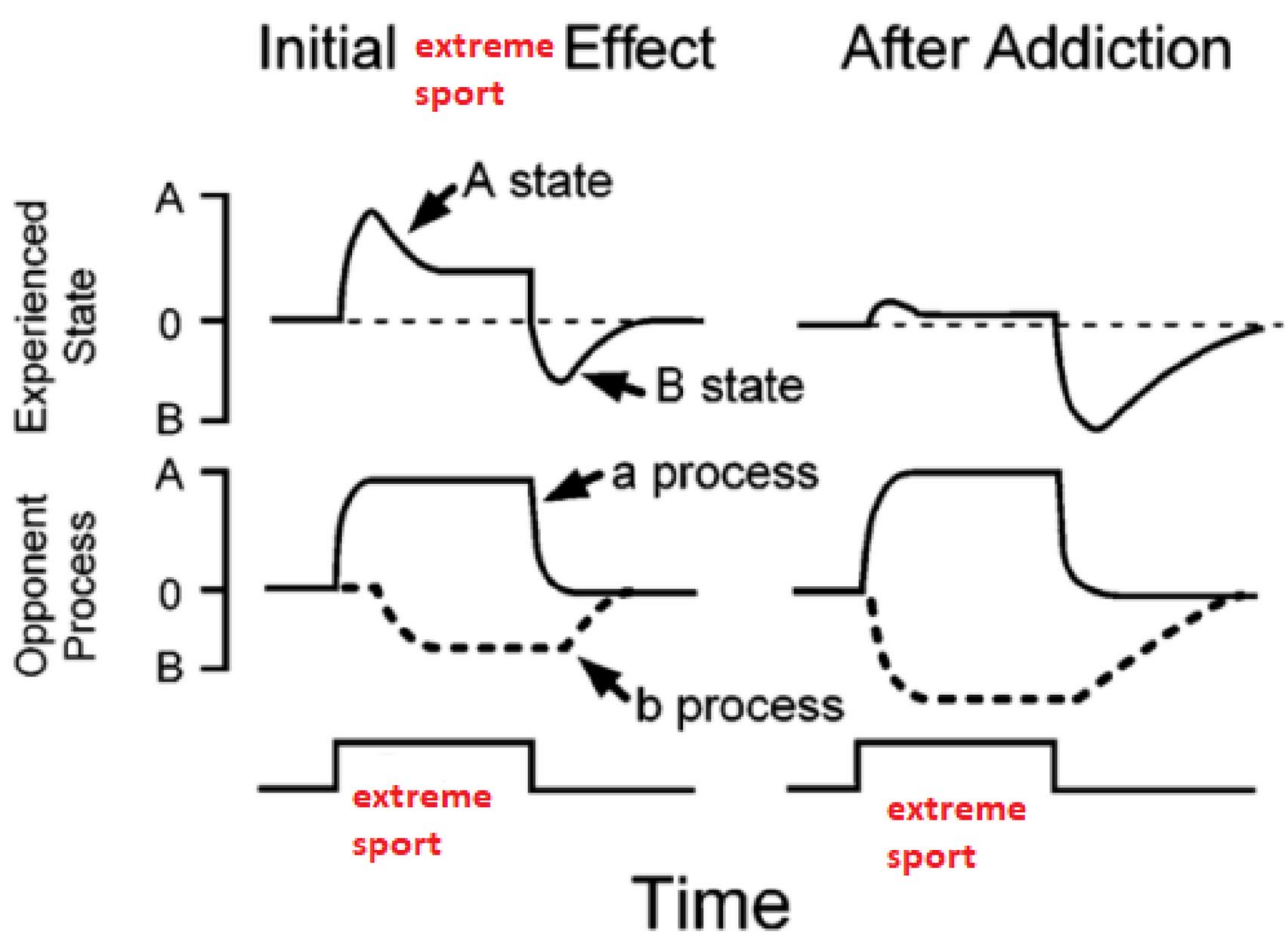
Theoretical background of the research, opponent process theory of addiction to extreme sports.

The utilization of empirical evidence, albeit limited, may serve as a methodology to circumvent potential biases in the comprehension of motivation and the addictive process in extreme sports. Roura Turet (2015) visualized the theory proposed by Solomon et al. and explained the transition processes to addiction with the aid of graphs (see Figure 1).

**Figure 2:**
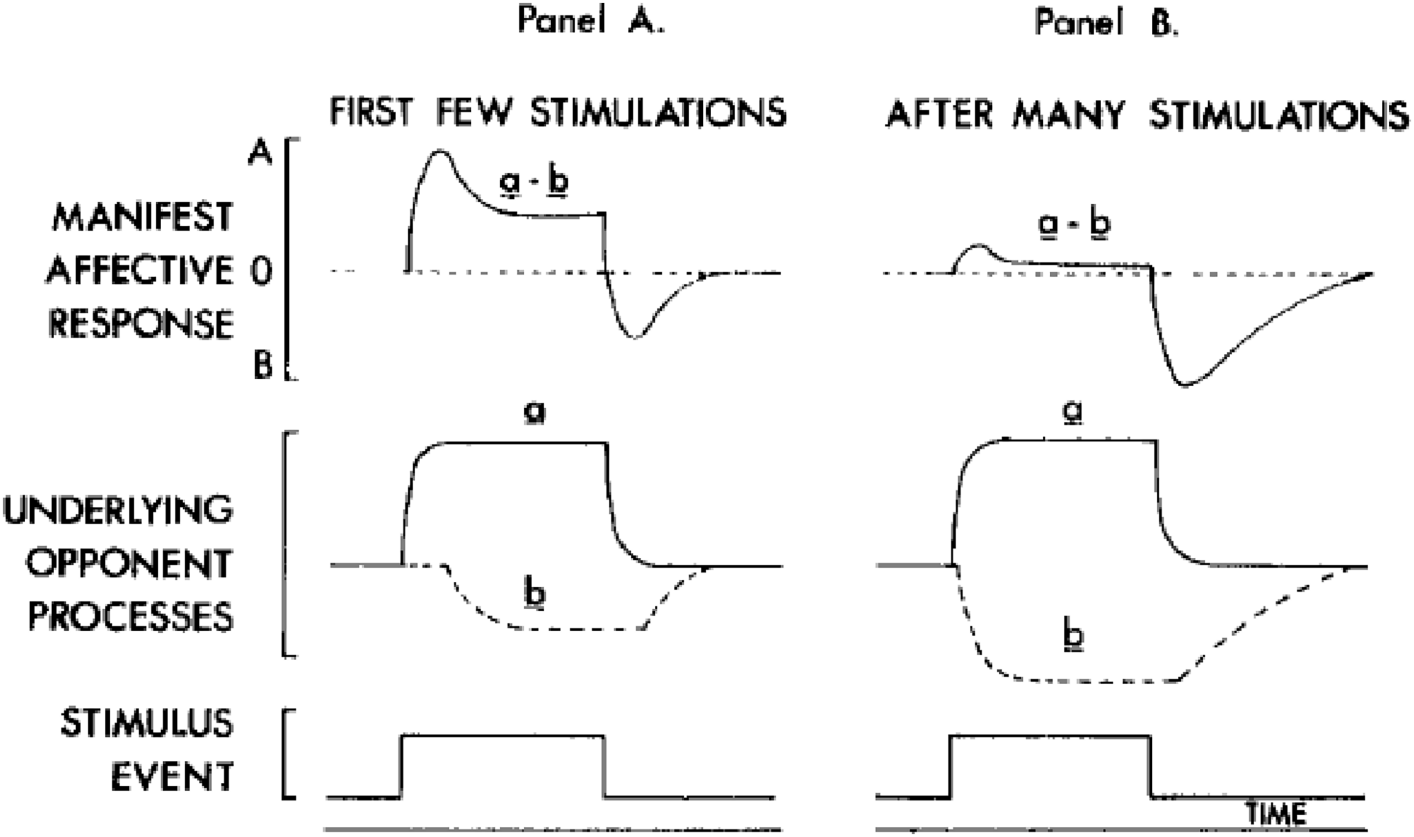
Solomon and Corbit (1974) opponent process theory.

As demonstrated in Figure 2, state A is initially large, followed by a smaller state B. It has been established that recurrent drug use leads to an augmentation in the magnitude and duration of the competing process, designated b, culminating in a substantial state B. The euphoric high experienced in state A is a direct consequence of the administered drug. Process b is characterized by a minor decline in the drug’s efficacy following the initial peak. In the event of subsequent administration of the drug, the B process is known to become more pronounced, manifesting as a tolerance to the drug’s euphoric effects. Therefore, the A state undergoes a reduction. In such cases, should the user wish to repeat their previous experience, it is necessary for them to increase the dose. In conclusion, given the assumption that the duration of the B process exceeds that of the A process, a distressing sensation of withdrawal is experienced upon the dissipation of the drug’s effects. Accordingly, the individual experiences the B state. It is hypothesized that process b will only be subject to alteration in instances of repeated drug intake. Indeed, this process is characterized by an increase in both size and duration, ultimately resulting in an experience dominated by unpleasant symptoms associated with withdrawal (Roura Turet, 2015).

Figure 2 shows the “Opponent Process Theory” proposed by Solomon and Corbit (1973), Figure 3 illustrates the addiction processes and the pathway to addiction as visualized by Roura Turet (2015), and Figure 1 depicts the theoretical background of the study (the relationship between substance addiction and extreme sports addiction) in a graph.

**Figure 3:**
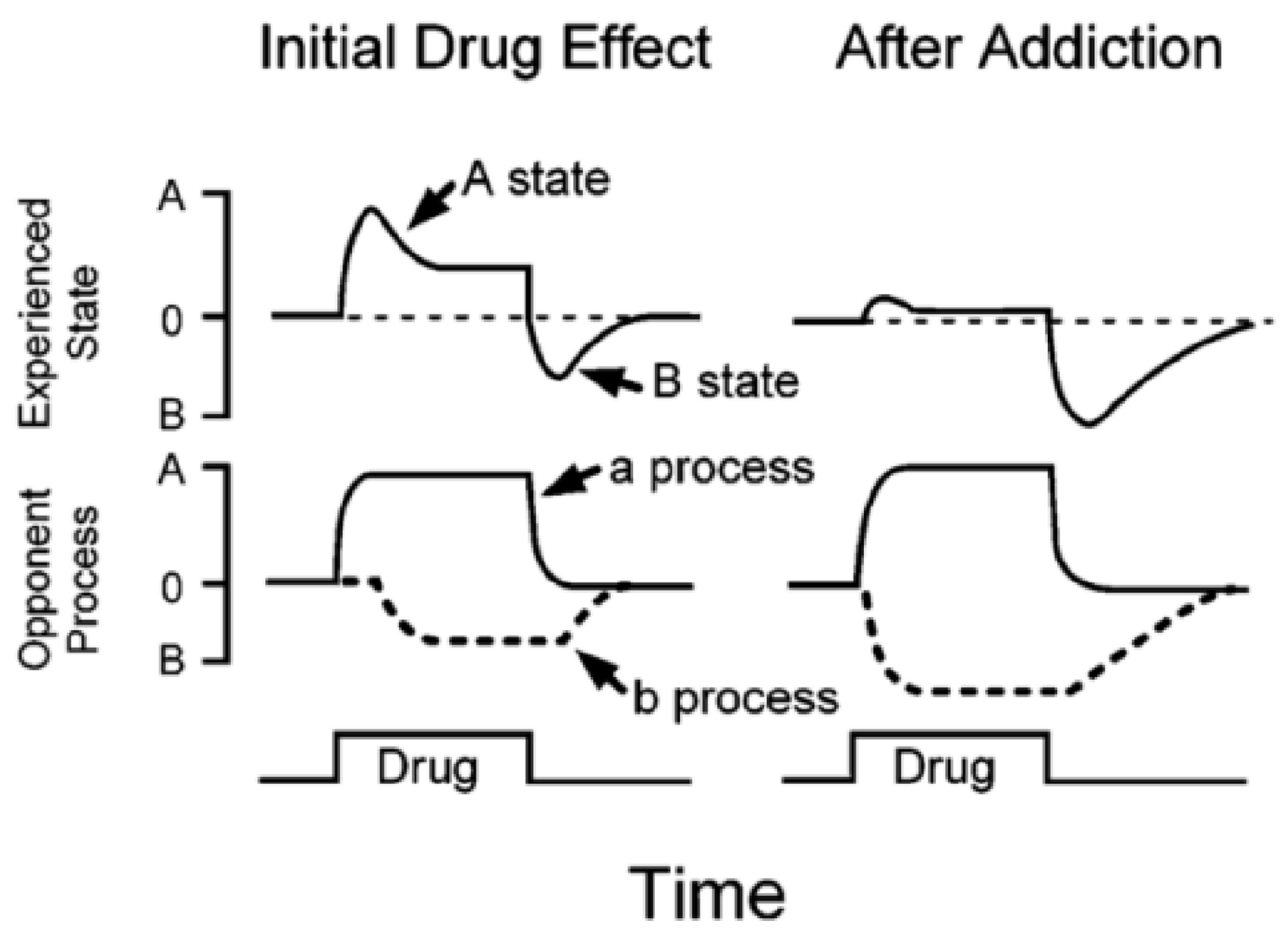
Roura Turet (2015) opponent process theory of addiction.

A review of recent studies on Opponent Process Theory and Current Approaches to Addiction in the literature reveals that this theory continues to play a central role in explaining the mechanisms of addiction. According to the Opponent Process Theory, the initial phase of substance use is characterized by the experience of positive affect and a sense of reward (a-process). However, over time, a negative affect (b-process) emerges in opposition to this initial response, gradually becoming the dominant emotional state. As a result, individuals begin to use substances not for enjoyment but to avoid negative feelings (Solomon and Corbit, 1974). At the neurobiological level, Koob and Le Moal (2008) expanded this model, demonstrating that an imbalance between dopaminergic reward systems and stress-related neuropeptide systems (e.g., CRF and norepinephrine) is decisive in the maintenance of addiction (Koob and Le Moal, 2008). In a similar vein, Ferrer-Pérez et al. (2024) comprehensive review underscores the notion that the Opponent Process Theory offers a substantial framework for elucidating tolerance, withdrawal, and conditioning processes. However, the review notes a deficiency in the theory’s capacity to sufficiently address individual variations and the heterogeneity of neuroadaptive processes. Greener and Storr (2023) conducted a comparative analysis of the opposing processes theory and various approaches to addiction, including the disease model and learning theories. Their findings indicated that the theory proved beneficial in elucidating emotional and motivational changes. However, the study also noted that the theory’s explanatory power at the neurobiological level remains constrained.

In their seminal 2021 study, Karin and colleagues proposed a novel physiological model (Karin et al., 2021). This model is based on opposing processes for endocrine changes occurring in the hypothalamic–pituitary–adrenal (HPA) axis, specifically in the context of alcohol addiction. Furthermore, the mathematical model developed by Chou and D’Orsogna (2022) formally demonstrated the reinforcement mechanism of addiction by numerically defining the dynamic interaction of the “a-process” and “b-process. When these findings are evaluated in general, the Opponent Process Theory provides a strong basis for explaining the continuation of addiction. However, it cannot provide a sufficient explanation on its own for the onset of addiction, individual sensitivities, and neurobiological diversity. All studies elucidate the reasons for the perpetuation of addiction. However, the explanatory power of these models is limited in terms of the ethology of addiction, its development in different individuals, and the role of individual differences. Contemporary approaches, notably neurobiological and computational models, seek to broaden the scope of the theory and enhance its comprehensiveness.”

## 2 Methodology

The study was conducted in eight stages, in accordance with the recommendations in the literature on scale development (DeVellis, 2003): The process is initiated with the determination of the structure. This is followed by the creation of the item pool and the preparation of the scale form. The next steps involve the assessment of face and content validity. The pilot application is then conducted, after which data is collected. The selection of items is the next stage, and this is followed by psychometric analysis. The final step is the evaluation of results.

### 2.1 Ethical approval

Permission for the study was obtained from the Scientific Research and Publication Ethics Committee of Mardin Artuklu University on April 15, 2025, with reference number 192089.

### 2.2 Participants

A global sample contains 1,073 English-speaking adventurers (Group 1: 583, 25.9% female, 74.1% male; Group 2: 490, 24.3% female, 75.7% male) was determined using the quota sampling technique, one of the non-probability sampling methods. The data was collected via an online survey link shared on social media platforms. This approach not only facilitated the participation process but also ensured the diversity of the sample on a global scale. Participants represented various countries, including the US, Germany, Australia, and others (see Table 1 for details). As demonstrated in Table 1, the participants hailed from various continents and regions, thereby enhancing the international representativeness of the study.

**Table 1:** Geographic distribution of participants (continents and regions).

Continent/Region	Countries (1st and 2nd sample groups)
Africa	Egypt: 17
Asia	India: 17, Indonesia: 17, Philippines: 17, Japan: 25, Korea: 17, Malaysia: 8, Singapore: 8, Thailand: 17
Europe – Western	Germany: 32, Austria: 27, Belgium: 16, Netherlands: 25, France: 32, Switzerland: 42
Europe – North	Denmark: 20, Estonia: 17, Finland: 25, Sweden: 25, Iceland: 20, Norway: 47, Latvia: 17
Europe – South	Italy: 27, Spain: 32, Portugal: 20, Greece: 20, Malta: 8
Europe – East	Bosnia and Herzegovina: 17, Bulgaria: 17, Czech Republic: 25, Hungary: 17, Poland: 25, Romania: 17, Russia (incl. Moscow): 25, Slovenia: 25
Europe – British Isles	United Kingdom (England, Scotland): 25, Ireland: 25
North America	United States: 47, Canada: 47
South America	Brazil: 42
Oceania	Australia: 47, New Zealand: 47
Middle East	Turkey: 30

A total of 1,073 participants from 43 countries are distributed across 11 regional groups. The distribution is based on the relative popularity of extreme sports in each country. This table shows how interest in extreme sports is related to a more developed sports culture, natural conditions, tourism infrastructure, and lifestyle trends.

According to the table, Oceania (Australia and New Zealand), North America (the US and Canada), and Northern Europe (especially Norway) have the most participants. Australia, New Zealand, US, Canada, and Norway each have the highest share with 47 participants. These countries are the world’s leading centres for nature-based extreme sports (e.g., bungee jumping, skydiving, rafting, MTB, skiing). Strong economic structures and high quality of life facilitate access to extreme sports. This shows that extreme sports are concentrated in countries with rich infrastructure and a focus on tourism.

On the other hand, Europe is the continent with the widest variety of participants. There are a total of 31 countries spread across the Western, Northern, Southern, and Eastern subgroups. Western Europe: Switzerland (42), France (32), and Germany (32) in particular have a high number of participants. The Alpine region (Switzerland, Austria, France, Italy) is the most intense area for extreme sports. Northern Europe: Norway (42), Finland (25), Sweden (25), and Iceland (20) stand out for their extreme natural conditions. Winter sports, ice climbing, and mountaineering activities are common in this region. Southern Europe: Spain (32) and Italy (27) are on the rise in extreme sports tourism. Sports such as diving, climbing, kite surfing, and mountain biking are popular around the Mediterranean. Eastern Europe: Participation rates are more balanced (between 17 and 25). This indicates that extreme sports are a developing market in the region.

Among Asian countries, Japan (25) has a higher participation rate than others. Japan’s developed sports culture and mountainous geography (especially for winter sports) have been influential. Participation numbers are relatively low in Southeast Asian countries (Malaysia, Singapore, Indonesia, Philippines, Thailand) (8–17). This can be explained by economic factors and the limited infrastructure for extreme sports.

For North America, the US and Canada are at the top of the list, each with 47 participants. These countries are the centre of world extreme sports events (X-Games, Red Bull Series, etc.). For South America, Brazil (42) is the continent leader due to its high natural potential (Amazon, Rio, Iguazu, kite surfing).

When evaluating the number of participants in other regions, the Middle East (Turkey – 30): Turkey has a moderate level of participation. It stands out in air sports and diving, particularly in the Cappadocia, Kas, and Fethiye regions. Africa (Egypt – 17): Participation is relatively low; this is because extreme sports are limited to tourist areas (e.g., Sinai, Sharm El Sheikh).

These distributions show that extreme sports are not evenly distributed globally, but are concentrated in higher-income, nature-oriented, and tourism-focused countries. Therefore, this picture clearly reveals not only the number of participants, but also the impact of lifestyle, climate, culture, and economic prosperity on interest in extreme sports.

Participants engaged in various adventure sports, high-risk sports, and extreme sports (e.g., base jumping, skydiving, wingsuit flying); categories were improved based on participant feedback and allowed for the selection of multiple sport categories (see Table 2 for details).

**Table 2:** Sports and categories practiced by participants.

Category	Adventure sports, high-risk sports, extreme sports
Airborne	Base jumping, skydiving, wingsuit flying, fun jumpers, hang gliders, paragliding
Involving ground movements	Parkour, free running, BMX, mountain biking/downhill biking, motorcycling
Winter sports	Skiing, backcountry skiing, heli-skiing, snowboarding, snowmobiling
Performed in water	Wakeboarding, jetpack, windsurfing, bodyboarding/big wave surfing, kiteboarding Canyoneering, rafting, white-water kayaking, scuba diving, cave diving, ice diving
Climbing and mountain activities	Mountaineering, traditional rock climbing, sport climbing, ice climbing, bouldering
Other	Bungee jumping, slackline walking, high-line walking, tightrope walking, caving, wild swimming, ice swimming

### 2.3 Scale development process

In order to ascertain the structure of MS and to establish the material pool, a comprehensive search of relevant databases was conducted. This included Google Scholar, Scopus, Web of Science, ProQuest, and SpringerLink The search strategy focused on studies pertaining to the themes of “addiction, behavioral addiction, motivation” (Ogden et al., 1997; Bachrach et al., 2012; Bohn et al., 1995; Hausenblas and Downs, 2002; Tiffany et al., 1993; Tiffany and Drobes, 1991; West and Roderique-Davies, 2008; Bohn et al., 1995; Vickers-Douglas et al., 2005). As demonstrated by Allen et al. (1998), Simons and Gaher (2005), McLain (2009) and Thomae et al. (2016), an examination was conducted of the works of Birtel and Wittemann (2016) and Schaler (1995). A thorough review of the extant literature revealed that the extant studies did not accurately reflect the theoretical basis that formed the starting point for the measurement tool to be developed. New items appropriate to the theory were therefore written for the measurement tool. Furthermore, during the item writing process, reference was made to experimental study reports (Epstein and Fenz, 1962, 1965; Solomon, 1980a, b; Solomon and Corbit, 1973, 1974;Solomon (1977); Roura Turet, 2015) and the studies addressed by Yavaş and Deveci (2025), which is the first pilot study on this theoretical basis. A new item pool was created.

Prior to the implementation of the measurement tool on the target group, a preliminary study was conducted on a sample of 60 individuals exhibiting comparable characteristics. This pilot study aimed to ascertain the clarity and comprehensibility of the items, as well as the duration of the application process. In this context, an item analysis was performed based on the participants’ feedback regarding the length of the items and items that were not fully understood, and items that reduced the reliability coefficient were removed, reducing the number of items from 53 to 22. In light of the specificity of the measurement instrument to be developed in the study, expert opinions were sought. However, the final decision on the items was determined by the researcher(s) themselves. This decision was made on the basis that the researcher(s) were the group best versed in the theoretical basis, and because experts in the field of measurement were part of the research team.

A 5-point Likert scale was devised for the scale. The scale is evaluated on a five-point rating scale ranging from “Never” (rating 1) to “Always” (rating 5). In the ensuing phase of the study, social media platforms (communities and branch-specific groups) with extreme sports participants from countries in Africa, Asia, Europe, South America, North America, and Oceania were accessed. Initially, a concise introduction accompanied by a visual representation of the survey was disseminated to the administrators and moderators of these platforms, with a request for their consent. Thereafter, the online survey link was disseminated in the groups that had granted permission, and participants were invited to click on the link and complete the survey. The process was subject to regular monitoring on a daily basis for a period of 3 months, and all queries raised by participants pertaining to the research were addressed individually. The data collection process was conducted meticulously, with constant communication maintained with the participants. As the development phase of the measurement tool was to be conducted in two distinct groups, the data collection phase was also divided into two stages. In this particular context, the initial phase of data collection was conducted for the purpose of Exploratory Factor Analysis (EFA). Subsequently, the data collection process was suspended for a period of two to three weeks, after which the Confirmatory Factor Analysis (CFA) data collection phase was initiated. A total of 1,073 data points were collected during the scale development process (First Group = 583; Second Group = 490).

### 2.4 Statistical analysis

The study comprises descriptive statistics for groups 1 and 2, as well as analyses related to the psychometric properties of the MS. The analysis includes descriptive statistics, an analysis of normal distribution, item analysis, exploratory factor analysis (EFA), confirmatory factor analysis (CFA), convergent and discriminant validity, measurement reliability and analyses of floor and ceiling effects. The analyses were performed using AMOS 23 for CFA, and Excel and IBM SPSS Statistics Version 20 for the remaining analyses.

## 3 Results

### 3.1 Item selection phase

Psychological variables are distinct from physical variables and possess a multidimensional structure. Thus, the factor analysis method is employed during the development process of measurement tools to reveal their psychological structure. Despite the prevalence of factor analysis as a preferred method in scale development studies, it is important to acknowledge the existence of alternative techniques, including correlation, t-test, and simple linear regression, which can be utilized in conjunction with factor analysis to ascertain the psychometric properties of a measurement tool (Asún et al., 2016; Boateng et al., 2018). The examination phase of the psychometric properties of the measurement tool commenced with item selection analyses. In the determination of the psychometric properties of the MS, the following item selection techniques were employed: correlation, factor analysis, and lower and upper 27% group techniques. In this direction, the correlation technique was initially employed. During the psychometric analysis process, although a lower bound of 0.30 for item–total correlations is frequently reported in the literature as an acceptable threshold (Worthington and Whittaker, 2006; DeVellis and Thorpe, 2021), more recent approaches in scale development recommend using values of 0.40 and above to obtain a clearer factor structure and higher measurement precision. Accordingly, in the present study, a cut-off value of 0.40 was adopted as a methodological choice to achieve a more coherent structure and to prevent ambiguous items from weakening the factor solution. As a result of the analyses, 6 items that did not meet this criterion were removed from the scale, and their exclusion did not adversely affect the theoretical coverage or measurement integrity of the instrument. At the end of this process, 16 items were retained for factor analysis.

Another item selection technique is the difference between group means of lower 27% and upper 27%. The technique employed involves the calculation of the difference between the lower 27% and the upper 27% group averages, with the resulting value being utilized to assess the discriminative power of the items in the measurement tool. Item discrimination is indicative of the extent to which the items in a measurement tool accurately reflect the feature under measurement. At this stage, it demonstrates the ability to distinguish each item from low and high levels (Boateng et al., 2018). The results of the analysis performed on the data from the first group (*n* = 583) indicate a 27% disparity between the upper and lower groups within the three-factor structure of the MS, at a significance level of *p* < 0.05.

### 3.2 Exploratory factor analysis (EFA)

In conclusion, the factor analysis technique was employed as a means of item selection in the present study. The MS factor structure (i.e., EFA) was examined on group 1 (*n* = 583) using the Principal Axis Factoring (PAF) method. The number of components to be removed was determined by examining the scatterplot in conjunction with the traditional Kaiser criterion (i.e., all factors with eigenvalues greater than one) (Büyüköztürk, 2011). The appropriateness of conducting EFA was substantiated by the sampling adequacy criteria of Kaiser-Meyer-Olkin (KMO = 0.797) and Bartlett’s Test of Sphericity (sd = 120, 5000.543, *p* < 0.0001). The analysis yielded three factors that collectively accounted for 63.00% of the total variance of the construct (see Table 3).

**Table 3:** Factor structure and factor loads of ms.

*n* = 583, Item number	Factor 1	Factor 2	Factor 3	Variance explanation of factors (%)
MS 24	0.891			% 63.00
MS 23	0.825			
MS 26	0.766			
MS 29	0.753			
MS 30	0.747			
MS 21	0.745			
MS 39		0.882		
MS 37		0.859		
MS 38		0.670		
MS 49		0.639		
MS 40		0.566		
MS 46		0.466		
MS 17			0.703	
MS 12			0.656	
MS 15			0.571	
MS 4			0.537	

Extraction method principal axis factoring.

### 3.3 Confirmatory factor analysis (CFA)

In order to validate the three-factor solution of the MS obtained with EFA, maximum likelihood CFA was applied using the robust standard error estimation method (MLR) with 16 MS items in Sample 2 (*n* = 490). For the close fit test, a chi-square *p*-value less than 0.05 was accepted for CFA fit goodness. Furthermore, a range of additional fit indices were measured. The following indices were measured: the Goodness-of-Fit Index (GFI), the Comparative Fit Index (CFI), the Tucker-Lewis Index (TLI), the Adjusted Goodness-of-Fit Index - Adjusted Goodness of Fit (AGFI), the Normed Fit Index (NFI), and the Standardized Error of Approximation Mean Squared (SAEM). In consideration of the aforementioned assumptions, the analyses of both first-order and second-order models yielded optimal model fit for MS (see Table 4 for details).

**Table 4:** First and second degree CFA results for MS.

Model	χ^2^/df	RMSEA	GFI	AGFI	IFI	NFI	TLI	CFI
First-order	2.20	0.07	0.88	0.83	0.92	0.87	0.91	0.92
Second-order	2.20	0.07	0.88	0.83	0.92	0.87	0.91	0.92

The fit indices for both first-order and second-order models fall within the limits accepted in the literature, thereby supporting the validity of the three-factor structure of the scale (Kline, 1994; Lomax, 2004; Schermelleh and Moosbrugger, 2003; Tabachnick and Fidell, 2007). This finding indicates that the factor structure obtained with EFA was robustly confirmed with CFA.

**Figure 4:**
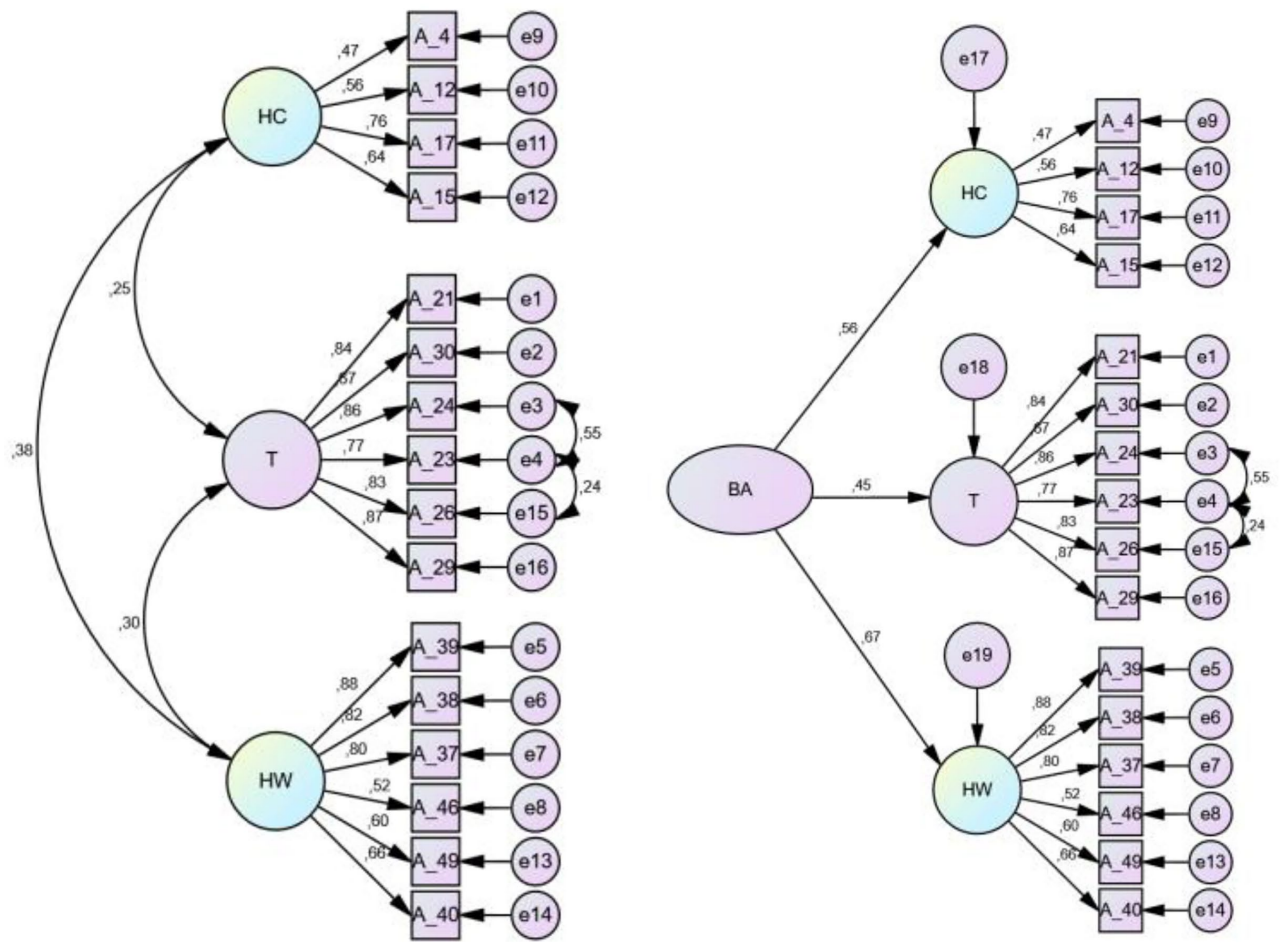
Graphical summary of CFA results obtained from the sixteen items of the MS on Sample 2 (*n* = 490).

Accordingly, the three-factor, 16-item structure of the MS was confirmed. In addition, the Path diagrams determined as a result of the CFA performed for the three-factor structure of the MS are shown in Figure 4.

### 3.4 Composite and discriminant validity

A further objective of this study was to conduct a more in-depth investigation into the validity of the MS. Subsequent to the validation studies pertaining to the structure of the MS, calculations were conducted with the assistance of the Excel programme to ascertain the composite and discriminant validity of the aforementioned structure. In order to ascertain the “combination validity” of the measurement tool, it is necessary to calculate the Combined Reliability-Composite Reliability (CR) and Average Explained Variance (AVE) -Average Variance Extracted (AVE) values. This can be achieved by calculating the factor loadings of each dimension with the help of the formula. In the extant literature, the interpretation of these values is contingent upon the AVE value exceeding 0.50 and the CR value surpassing 0.70. Conversely, in order to ascertain the “discriminant validity” of the measurement tool, it is necessary to calculate the Square Root of the Average Explained Variance (√AVE) value and the correlation value between the factors. Discriminant validity is said to be ensured if the values obtained by taking the square root of the average explained variance (AVE) for each dimension are greater than the correlations with the other dimensions (Hair et al., 2009; Fornell and Larcker, 1981; Kline, 1994). At the same time, if the limit value is not above 0.90, this is also said to be indicative of discriminant validity (Fornell and Larcker, 1981; Kline, 1994). In this context, the Average Explained Variance (AVE), the Square Root of the Average Explained Variance (√AVE), and the Combination Confidence (CR) values were also calculated.

The AVE value of the tolerance subscale of MS was calculated as 0.65, the√AVE value as 0.80, and the CR value as 0.91. The AVE value of the hedonic contrast subscale of MS was calculated as 0.37, the√AVE value as 0.60, and the CR value as 0.86. The AVE value of the hedonic withdrawal subscale of MS was calculated as 0.52, the√AVE value as 0.72, and the CR value as 0.86. As stated in the relevant literature (Hair et al., 2009), the Average Variance Extracted (AVE) should be greater than 0.50, and the Cronbach’s Alpha (CR) should be greater than 0.70. The study revealed that the Average Variance Equations (AVE) value was less than 0.50. Fornell and Larcker (1981) proposed a guideline stipulating that, in instances where the AVE value is below 0.50, the AVE values can be accepted if the CR value is above 0.70. Despite the fact that the AVE values for these two sub-dimensions were below 0.50 in the study, the reference values were taken into account because the CR values were above 0.70 and the AVE values were also accepted. The findings indicate that the measurement model fulfills the criteria for composite and discriminant validity.

### 3.5 Measurement reliability

The reliability of the MS was assessed using Cronbach’s Alpha and Split-Half methods. According to the results of the Cronbach Alpha analysis, the tolerance subscale of the MS yielded a value of 0.92, the hedonic contrast subscale registered 0.70, and the hedonic withdrawal subscale attained 0.85. In the literature, values exceeding 0.70 are indicative of reliable measurement tools (Büyüköztürk, 2011). In addition, the evaluation employing the Split-Half method yielded both Spearman-Brown and Guttman values of 0.87, thereby signifying the scale’s exceptional reliability (refer to Table 5 for specifics). The findings indicate that the subscales of the MS and the overall measurement tool can be used reliably.

**Table 5:** Cronbach alpha and split-half reliability values, item numbers, and contents of the sixteen-item MS.

Subscales	Item numbers and contents	Cronbach Alpha (α)	Spearman-Brown	Guttman
MS	Hedonic Contrast (HC)	I cannot wait to make the experience happen.	0.70	0.87	0.87
		Immediately after the experience, I usually react like shouting or screaming.			
		After a little bit of experience, the constant urge to talk comes.			
		After a little bit of experience, I become too energetic to sit still.			
	Tolerance (T)	Experiencing the same difficulty all the time makes my activity mundane.	0.92		
		Experiencing the same place all the time makes me lose my excitement after a while.			
		Experiencing the same place all the time makes me lose my enthusiasm after a while.			
		Experiencing in the same place reduces my level of pleasure.			
		Experiencing the same level of difficulty lowers my level of pleasure.			
		Experiencing the same level of difficulty causes me to alienate from the activity.			
	Hedonic Withdrawal (HW)	I get stressed when I cannot perform the experience.	0.85		
		I worry more when I cannot perform the experience.			
		I feel more nervous when I cannot carry out the experience.			
		I live the experience with the thought that I will be unhappy if I cannot perform the experience.			
		I become aggressive when I am unable to perform the experience.			
		When I am not able to perform the experience, I feel empty.			

### 3.6 Analysis of the distribution: floor and ceiling effects

The floor and ceiling effects of the MS were also examined. The floor effect is defined as the proportion of individuals who responded “never” (1 point) to all items and accumulated a total of sixteen points. Conversely, the ceiling effect denotes the proportion of individuals who responded “always” (5 points) to all items and accumulated a total of eighty points. As Pontes and Griffiths (2015) assert, a ratio exceeding 15% indicates the presence of a floor and ceiling effect, a phenomenon that is considered to be undesirable. Upon analysis of the distribution of the obtained scores, it was observed that a total of 12 individuals (2.4%) attained the maximum score of 80 points, while no participants (0%) achieved the minimum score of 16 points. This finding indicates the absence of a floor and ceiling effect in the distribution of scores.

### 3.7 MS scoring

To identify exercise addiction and other types of addiction, criteria based on the Diagnostic and Statistical Manual of Mental Disorders, Fourth Edition (DSM-IV), as well as criteria for other substance addictions, are used (Hausenblas and Downs, 2002). Individuals were assessed using the DSM-IV to determine whether they fell into the non-addicted symptomatic (showing symptoms) or non-addicted asymptomatic (not showing symptoms) categories, both of which carry a risk of behavioral addiction. This assessment is based on the criteria of hedonic antagonism, tolerance and hedonic deprivation. Consisting of 16 items and three subscales, the MS was developed based on the OPS and provides two main pieces of information.

#### 3.7.1 Average scores for behavioral addiction symptoms

Classification of individuals into three categories: (a) individuals with behavioral addiction; (b) non-addicted symptomatic individuals; and (c) non-addicted asymptomatic individuals.

ROC (Receiver Operating Characteristic) analysis was applied to determine the cut-off point for MS. In the context of ROC analysis, the discriminatory power of a test is evaluated based on the area under the curve (AUC) value. According to Saunders and Trapp (1994), a value of 0.5 indicates no discriminatory power, the range 0.5–0.7 indicates insignificant discriminatory power, the range 0.7–0.8 indicates acceptable discriminatory power, the range 0.8–0.9 indicates good discriminatory power, and values above 0.9 indicate excellent discriminatory power. The analysis results demonstrated that the area under the curve (AUC) value for MS was 1.000, thus indicating excellent discriminatory power. This finding indicates that the MS can distinguish between asymptomatic and non-symptomatic non-dependent individuals and behaviourally dependent individuals with 100% accuracy. The cut-off point analysis determined that the cut-off point for the scale is 48.5. Consequently, individuals scoring below 48.5 were classified as asymptomatic non-addicts, those scoring 48.5 were classified as symptomatic non-addicts, and those scoring above 48.5 were classified as behavioral addicts. The findings definitively confirm the scale’s capacity to demonstrate both high sensitivity and specificity in assessing the risk of behavioral addiction in MS, and it can therefore be said to be a reliable scale.

## 4 Discussion and conclusion

In extreme sports, nature is seen as the perfect arena in which participants can demonstrate their individual capabilities. However, in contrast to this mutual struggle between extreme athletes and nature, nature is generally accepted as a separate entity from humanity. This is far from the reality in which nature is perceived as an inanimate object. This is because extreme athletes perceive nature as something other than an inanimate object. They accept the relationship between humans and nature as a bond and view these experiences as a kind of addiction (Storry, 2003; Pain and Pain, 2005; Salassa and Zapala, 2009; Brymer and Gray, 2010; Brymer and Schweitzer, 2013).

Based on OPT, this study followed a literature-supported scale development process to create a multidimensional MS that reflects the experiences of extreme athletes and addresses the concepts of motivation and addiction. The results revealed a three-factor solution using the 16 items of the MS. This structure emerged in EFA and was subsequently confirmed by CFA results. These results provided fit indices confirming the applicability of the proposed three-factor solution, as the model showed an optimal fit to the data. AVE, CR and √AVE values were calculated to assess the composite and discriminant validity of the MS. The results showed that each dimension of the measurement model measured a distinct construct, ensuring discriminant validity. Cronbach’s alpha and split-half reliability results also proved that the measurement tool was reliable. All results confirmed that the MS is a valid and reliable measurement tool. The MS consists of a five-point Likert scale with 16 items and three dimensions. In this context, responses to each item in the measurement tool are ranked as ‘Never’, ‘Rarely’, ‘Sometimes’, ‘Often’, or ‘Always’; 1, 2, 3, 4, or 5.

The findings of this study demonstrate that the three-factor structure reflects a complex psychological process underlying participation in extreme sports (Martinho et al., 2024). This structure indicates that such experiences cannot be explained solely through physical risk-taking or adrenaline-seeking perspectives, but rather through emotional regulation and internal motivation mechanisms. The identified dimensions suggest that extreme sport participation involves cyclical emotional dynamics in which high affective arousal is followed by a strong desire to restore psychological balance (Woodman et al., 2010; Wagstaff, 2014). The reports of discomfort, tension, or emotional emptiness when participation is interrupted highlight behavioural-addiction-like tendencies, while the increasing need for greater stimulation supports the notion of escalating engagement over time. These results indicate that motivation in extreme sports is multidimensional, shaped by internal rewards, personal identity construction, self-efficacy, and psychological fulfillment (Claes et al., 2025; Heirene et al., 2016; LaRocque and Moreau, 2022). Therefore, the measurement tool developed in this study provides a valuable framework for objectively assessing these experiences, contributing to a deeper theoretical understanding of motivation and behavioral addiction in extreme sports and offering a foundation for future empirical research.

The extant literature on the subject of behavioral addiction in extreme sports is limited. This is due to the fact that behavioral addiction has been the focus of predominantly qualitative studies, and the concept itself is a recent development. Accordingly, this study makes a significant contribution to the field of extreme sports literature by providing a novel, concise and valid psychometric tool for the assessment of motivation and behavioral addiction. Future researchers in this field are advised to utilize the measurement tool to examine variations across different populations. Moreover, the utilization of the MS will facilitate a comparative analysis of the experiences of extreme sports participants, thereby enhancing our understanding of both the similarities and differences that exist across a range of activities.

The MS plays a pivotal role as it is the inaugural measurement tool study in the international literature. This study makes a significant contribution to the existing body of research on addiction and motivation, offering both theoretical insights and a novel measurement tool. Firstly, it is important to acknowledge that previous studies have exclusively focused on qualitative research. Consequently, this study makes a significant contribution to the existing literature on motivation and addiction by developing a theory-based measurement tool that explores how individuals participating in extreme sports can become addicted to such activities.

This study has some limitations. Although the sample size was large (1,073 participants) and the sample generally represented the population, reaching more participants could have increased the generalisability of the findings. It is recommended that future studies aim to validate the current findings using more representative samples. Furthermore, participants’ experiences could be reflected more comprehensively by designing mixed-methods studies that use both qualitative and quantitative methods. Examining the diversity of behavioral addiction experiences by considering different groups or subgroups would also contribute to future research. Overall, this study’s findings empirically support the concept of behavioral addiction, demonstrating that the developed measurement tool can validly and reliably inform research in this field.

### 4.1 Theoretical and methodological implications

The present study developed an MS, OPT-based three-factor structure: Tolerance, Hedonic Antagonism, and Hedonic Withdrawal. The scale’s structural validity is supported by EFA and CFA analyses, which demonstrate that the subscales reflect the emotional processes of OPT (see Tables 3, 4). The following three factors provide a theoretical framework for understanding the motivation and behavioral addiction tendencies of extreme sports participants.

The existing body of literature on the subject of motivation and behavioral addiction in the context of extreme sports is limited. The primary rationale for this is that behavioral addiction is a relatively recent phenomenon, which has been addressed principally in conceptual terms in qualitative studies. The MS developed in this study addresses this lacuna by providing a valid and reliable psychometric instrument for the extant literature on extreme sports, high-risk sports, and adventure sports.

The contributions of the MS are as follows:

- Contribution to the understanding of behavioral addiction: The extent to which participants exhibit addiction tendencies can be ascertained through the analysis of their experiences in extreme sports.
- Challenging Stereotypes: The scale provides an opportunity to test and question stereotypical judgments about extreme sports participants, such as “adrenaline junkie” or “risk seeker.”
- The present study explores the compatibility of the subject with OPT. The scale strengthens the theoretical basis by measuring three dimensions and the general tendency within the OPT framework.

It is recommended that future research efforts focus on a comparative analysis of the experiences of extreme sports participants, utilizing the MS in diverse populations. This approach will facilitate a more profound comprehension of the similarities and differences across different sporting activities. In this regard, the MS offers a significant theoretical contribution as a novel and original instrument for quantitative research in the field.

## Data Availability

The raw data supporting the conclusions of this article will be made available by the authors, without undue reservation.
